# A Survey Assessment of Neurosurgeons’ Interest in Osteopathic Medicine and Its Integration Into Their Practice

**DOI:** 10.7759/cureus.55707

**Published:** 2024-03-07

**Authors:** Devin W Kolmetzky, Dillon B Gooder, Evan S Polly, Sarah N Glisan, Zein Al-Atrache, Clint A Badger, Steven S Yocom, Alan R Turtz, Donald L Allison

**Affiliations:** 1 Department of Osteopathic Medicine, Philadelphia College of Osteopathic Medicine, Philadelphia, USA; 2 Department of Neurosurgery, Cooper University Hospital, Camden, USA

**Keywords:** survey, research interest, osteopathic research, neurosurgery, osteopathic medicine

## Abstract

Introduction: Osteopathic manipulative medicine (OMM) encompasses techniques guided by the tenets of osteopathy aimed at facilitating the body's natural self-healing capabilities as a treatment option for injury or illness. This approach recognizes the interrelationship of structure and function in promoting overall health. The clinical applications of OMM have been highly researched throughout different subspecialties of medicine; however, there is a notable lack of osteopathic-based research targeted toward neurosurgical patient populations.

Methods: This cross-sectional descriptive study was conducted via a survey generated using SurveyMonkey (SurveyMonkey, San Mateo, CA, USA; accessed at www.surveymonkey.com). Subjects for this survey were gathered using a convenience sampling method in which emails of all neurosurgeons listed in the "Member Directory" on the American Association of Neurological Surgeons website were compiled into a mailing list. The survey was sent to all 6,503 emails collected, and the responses were recorded over the next month. The responses for each survey question were averaged and, when appropriate, compared using a two-tailed T-test, with statistical significance defined as a p<0.05. Where applicable, simple linear regression analysis was used to assess correlations between survey data. The measured outcomes included neurosurgeons' (1) knowledge of and (2) attitudes toward OMM.

Results: Both MD and DO neurosurgeons reported using OMM (or referring their patients for OMM) less than once per year. In comparison to their MD colleagues, neurosurgeons carrying a DO degree ranked their familiarity with the tenets of osteopathic medicine (p<0.0001) and their knowledge of the applications of OMM in their practice (p=0.0018) significantly higher. Greater reported familiarity with the tenets of osteopathic medicine and applications of OMM showed a positive correlation with neurosurgeons' comfort in recommending OMM as a nonsurgical, preoperative treatment option, as a post-surgical, rehabilitative treatment option, and as a pain management option (p<0.0001 for all). There was a clear interest in seeing further osteopathic-based neurosurgery research by both MD and DO neurosurgeons, as well as a trend of interest in incorporating OMM into their practice if shown to be clinically beneficial.

Conclusions: Both MD and DO neurosurgeons are interested in seeing more research into the applications of OMM in their patient populations and, most importantly, are likely to integrate OMM into their practice if presented with research detailing clinical benefits to their patients. This study highlights the clinical interest of neurosurgeons in further research into the applications of OMM specific to the field of neurosurgery.

## Introduction

The core principles underlying the practice of osteopathic medicine are the facilitation of the body’s natural self-healing capabilities and the promotion of homeostasis [1,2]. This approach to treatment is defined by four major tenets: (i) the body is a unit of body, mind, and spirit; (ii) the body is capable of self-regulation, self-healing, and health maintenance; (iii) structure and function are reciprocally interrelated; and (iv) rational treatment is based upon an understanding of the basic principles of body units, self-regulation, and the interrelationship of structure and function [3]. Osteopathic medicine aims to treat somatic dysfunction, defined as an impaired or altered function of related components of the body's framework system, including skeletal, arthrodial, and myofascial structures, and their related vascular, lymphatic, and neural elements [2,4]. Somatic dysfunction itself is characterized by (i) tenderness to palpation; (ii) static structural asymmetry; (iii) reduced range of motion; and (iv) tissue texture changes [1,5].

One way in which osteopathic physicians can apply the principles of osteopathic medicine in their practice is through the application of hands-on, manually guided forces in a manner collectively termed osteopathic manipulative medicine (OMM) [4]. OMM has widely diverse applications throughout different fields of medicine and has been researched in an extensive range of clinical scenarios [1,2,5-12]. However, a literature search related to applications of osteopathic medicine, specifically in neurosurgical patient populations, yields relatively few results. In a search of PubMed at the time of this study’s submission, 10,730 articles contained keywords related to osteopathic medicine, but of these, only 14 were research related to the care of patients with conditions typically requiring neurosurgical consultation [13-26].

Despite this limited volume of osteopathic-based neurosurgery research, there is existing literature exploring topics related to the realm of the nervous system and other pertinent medical domains. There is a particular abundance of information related to the use of techniques like counterstain, muscle energy, and HVLA in the management of chronic, non-specific cervical neck [9,10,12] and lower back pain [11,18,27-32], as well as in the management of postoperative pain [33-35]. Though subsets of these research topics may be applicable to neurosurgical patients, little research is to be found on the use of OMM specifically in this patient population [36,37]. Rib raising has been shown to influence the balance of the autonomic nervous system [38,39]. Craniosacral therapy has been shown to alleviate symptoms in individuals who have suffered a concussion [40-42] and in individuals with normal pressure hydrocephalus [17]. The glymphatic system - the lymphatic drainage system of the CNS - is shown to be modifiable with cranial osteopathic manipulative techniques as well as Dalrymple and thoracic pump techniques in the setting of traumatic brain injury [13,15].

Among all medical subspecialties, the neurosurgical community is one of the most prolific publishers of self-evaluation and outcome research [43]. With this commitment to evidence-based patient management and in light of the body of OMM research, it is of particular interest why so little neurosurgery-focused OMM research is published. Given the neurosurgical community’s dedicated culture of research, it is questioned if neurosurgeons are interested in seeing more osteopathic-based research aimed specifically at the management of their patient populations and are interested in applying said research into their clinical practice. This article was previously presented as an abstract at the 2023 Philadelphia College of Osteopathic Medicine Student Research Day on May 5, 2023, and as a podium presentation at the 2023 American College of Osteopathic Surgeons Annual Clinical Assembly on September 23, 2023 [36,37].

## Materials and methods

Study design

This study is an exploratory cross-sectional survey created by four osteopathic medical students at the Philadelphia College of Osteopathic Medicine. Questions in this survey aimed to better understand US neurosurgeons’ current utilization rates, perceptions, and interests in osteopathic medicine, as well as their desire to integrate osteopathic-based research into practice. The survey was approved first by the home institution’s course director for osteopathic medicine, followed by two DO neurosurgery residents and both DO and MD neurosurgery attendings from a separate institution (Cooper University). The contents of this survey are listed in Table 1.

**Table 1:** List of survey questions

Survey question	Response options
Q1: What is your age	Free response
Q2: What is your gender?	Male, female, prefer not to answer, other
Q3: What type of medical degree do you hold?	MD, DO
Q4: What year did you graduate medical school?	Free response
Q5: In which state(s) did you attend medical school?	Free response
Q6: In which state(s) did you do your residency?	Free response
Q7: In which state(s) do you currently practice?	Free response
Q8: How would you describe your current practice?	Hospital-based, group practice, private practice, other
Q9: About how often do you use, or refer a patient for, osteopathic manipulative medicine in your practice?	1=never, 2=once per year, 3=once per month, 4=once per week, 5=every day
Q10: On a scale of 1-5, what is your familiarity with the tenets of osteopathic medicine?	1=no knowledge; 2=unfamiliar; 3=somewhat familiar; 4=familiar; 5=very familiar
Q11: On a scale of 1-5, what is your familiarity with applications of osteopathic manipulative techniques in your practice?	1=no knowledge; 2=unfamiliar; 3=somewhat familiar; 4=familiar; 5=very familiar
Q12: On a scale of 1-5, how comfortable are you recommending osteopathic manipulative medicine as a non-surgical, preoperative option (in general) for your patients?	1=very uncomfortable; 2=uncomfortable; 3=somewhat comfortable; 4=comfortable; 5=very comfortable
Q13: On a scale of 1-5, how comfortable are you recommending osteopathic manipulative medicine as a post-surgical, rehabilitation option (in general) for your patients?	1=very uncomfortable; 2=uncomfortable; 3=somewhat comfortable; 4=comfortable; 5=very comfortable
Q14: On a scale of 1-5, how comfortable are you recommending osteopathic manipulative medicine as a pain management option (in general, both pre- and post-operatively) for your patients?	1=very uncomfortable; 2=uncomfortable; 3=somewhat comfortable; 4=comfortable; 5=very comfortable
Q15: On a scale of 1-5, how interested are you in seeing more research into potential applications of osteopathic manipulative medicine in the field of neurosurgery?	1=no interest; 2=uninterested; 3=somewhat interested; 4=interested; 5=this must happen
Q16: On a scale of 1-5, if you were presented with peer-reviewed research showing positive results from osteopathic manipulative techniques used in neurosurgical patient populations, how likely are you to integrate their use into your own practice?	1=I would never; 2=very unlikely; 3=unlikely; 4=likely; 5=very likely
Q17: For the previous question, if you answered "I would never," "very unlikely," or "unlikely," what barriers do see that prevent you from doing so?	Free response
Q18: FOR DOs: Have you used osteopathic manipulative techniques in your practice? If so, please comment on the techniques you use, the patient population you use them in, etc.	Free response

Participants

This study was approved by the Institutional Review Board of the Philadelphia College of Osteopathic Medicine (protocol number: H23-029X). An electronic mailing list was created in a convenience sampling fashion using contact information compiled within the "Member Directory" on the American Association of Neurological Surgeons (AANS) website (www.aans.org). Members were screened by "member type" for inclusion or exclusion in this study. Membership types meeting inclusion criteria for this study were (i) fellows (FAANS), (ii) candidates (residents/fellows), (iii) provisional, (iv) lifetime, (v) associates (MDs and PhDs), (vi) international candidates, and (vii) international neurosurgeons. Membership types meeting exclusion criteria for this study were (i) advanced practitioners (RNs, NPs, and PAs), (ii) medical students, (iii) allies (admin and surgical tech), (iv) honorary, and (v) affiliates. In total, 6,503 subjects were selected from the AANS "Member Directory" based on their "member type" status. Furthermore, survey respondents who did not provide all requested demographic information, did not complete all applicable survey questions, and are currently retired were also excluded from this study.

Survey

This is a descriptive study delivered via a survey generated using SurveyMonkey (SurveyMonkey, San Mateo, CA, USA; accessed at www.surveymonkey.com). The survey was distributed anonymously via email using the generated electronic mailing list described above. The initial email provided a brief explanation of the study being conducted and a web link to complete the survey. The survey was left open for one month after the first email was sent. At the three-week mark, a second email was sent to remind participants of the survey and its end date. Participants were prompted in both emails to respond if they wished to be removed from the study. The authors of this study were also included on the mailing list in order to access the survey and input mock data to ensure its functionality.

Statistics

Data cleaning was manually performed on all collected survey responses. Respondents who did not answer all questions of the survey were excluded from statistical analysis. Statistical analysis was performed using Prism v9.5.1 (GraphPad Software, La Jolla, CA, USA). The independent variables include self-reported knowledge and familiarity with the tenets and applications of osteopathic medicine (on a scale of 1-5). The dependent variables include self-reported rates of neurosurgeons’ use of OMM in practice, their comfort in recommending OMM for their patients, their interest in seeing further research into applications of OMM in neurosurgical patients, and their interest in integrating OMM in their practice if presented with research demonstrating a clinical benefit to their patient population (also on scales of 1-5). Responses from MDs and DOs were averaged and, when appropriate, were compared using a two-tailed T-test, with statistical significance defined as a p<0.05. Where applicable, simple linear regression analysis was used to assess correlations between survey data.

## Results

Of the 6,503 neurosurgeons emailed, 133 submitted a survey response, a response rate of 2.05%. Of these, 14 were excluded from the study for the following reasons: one respondent did not provide any demographic information, nine respondents did not complete the survey responses, and four respondents are currently retired. In total, data from 119 participants was included in the data analysis. The average time to complete the survey was two minutes and 43 seconds. The demographic data for the study participants is listed in Table 2.

**Table 2:** Survey question data (questions 1-8) The number of survey respondents is represented by "N," with the percent of each subgroup in relation to the total number of survey respondents represented beside it (%). The mean of each metric is listed in the second column from the right. The standard deviation (SD) is reported in the right-most column.

Demographic information	Demographic metric	N (%)	Mean	SD
Total respondents	-	119	N/A	N/A
Gender	-	-	-	-
-	Male	93 (78.15%)	N/A	N/A
-	Female	21 (17.65%)	N/A	N/A
-	Prefer not to answer	5 (4.20%)	N/A	N/A
Degree type	-	-	-	-
-	MD	112 (94.12%)	N/A	N/A
-	DO	7 (5.88%)	N/A	N/A
Age	-	-	-	-
-	≤39 years old	44 (36.97%)	33.48	3.04
-	40-59 years old	50 (42.02%)	49.00	6.21
-	≥60 years old	25 (21.01%)	67.48	7.02
-	Total	119	47.14	13.72
Type of practice	-	-	-	-
-	Hospital-based	75 (63.03%)	N/A	N/A
-	Private practice	17 (14.29%)	N/A	N/A
-	Group practice	17 (14.29%)	N/A	N/A
-	Academic, university setting	9 (7.56%)	N/A	N/A
-	Consulting	2 (1.68%)	N/A	N/A

**Figure 1:**
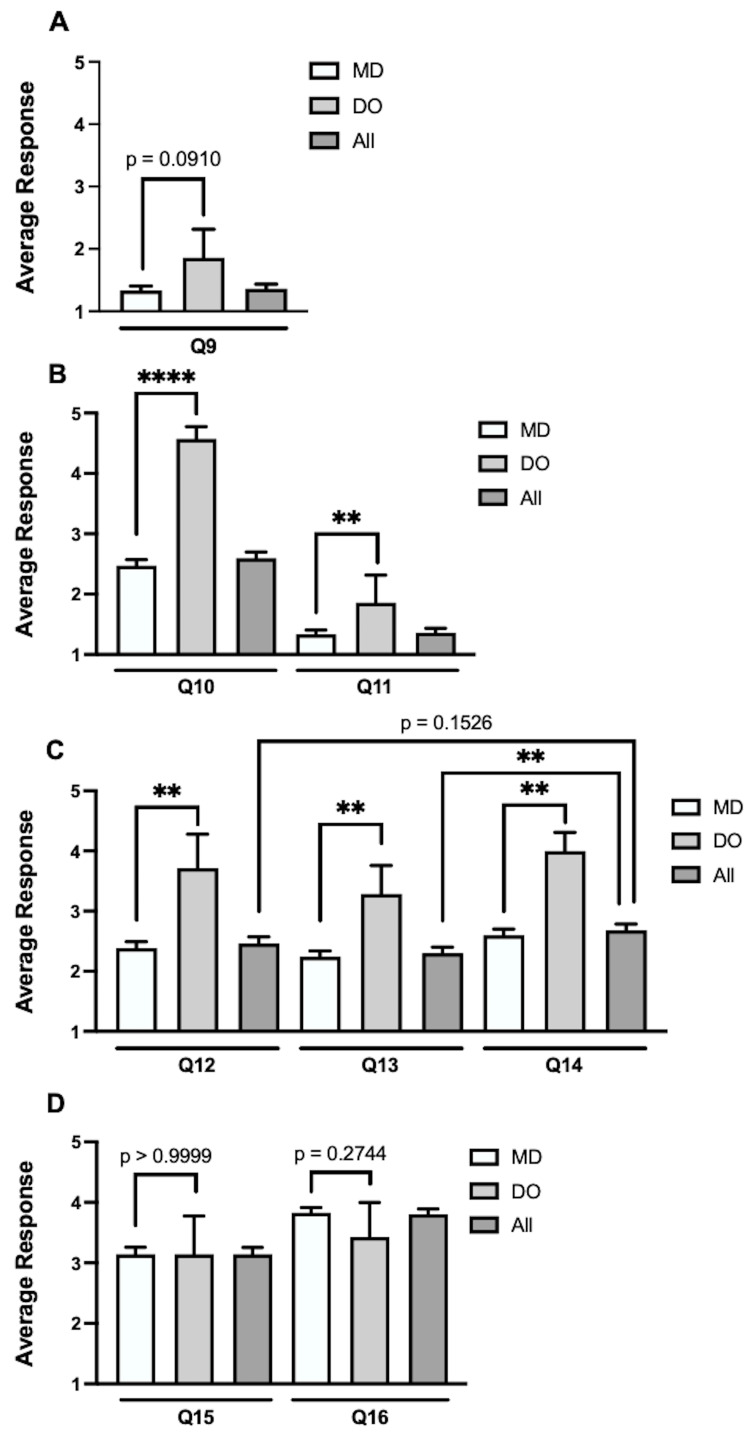
Comparisons of survey data between MDs and DOs (A) Both MDs and DOs reported using OMM or referring their patients for OMM less than once per year. DOs trended toward higher rates than MDs. (B) DOs rated both their familiarity with the tenets of osteopathic medicine and their familiarity with applications of OMM higher than MDs. (C) DOs reported greater levels of comfort than MDs in recommending OMM as a nonsurgical preoperative option, as a post-surgical rehabilitation option, and as a pain management option. (D) MDs and DOs both showed interest in seeing more osteopathic-based research focused toward neurosurgical patients, as well as interest in applying OMM into their practice if provided with research that proves a clinical benefit to their patients. Statistical significance was defined as p<0.05.

Both MD and DO neurosurgeons reported using OMM or referring their patients for OMM less than once per year. DO neurosurgeons trended toward referring patients more than MDs (DO average = 1.86 ± 1.22 and MD = 1.33 ± 0.76, p=0.0910, where "1" = "never" and "2" = "once per year"), with 78.99% responding "never" (Figure 1A). In comparison to their MD colleagues, neurosurgeons carrying a DO degree ranked their familiarity with the tenets of osteopathic medicine (averages, DO = 4.57 ± 0.53 and MD = 2.47 ± 1.03, p<0.0001) and their knowledge of the applications of OMM in their practice (averages, DO = 3.29 ± 1.38 and MD = 1.96 ± 1.05, respectively, p=0.0018) significantly higher (Figure 1B).

Respondents (combined MDs and DOs) were more comfortable recommending OMM as a nonsurgical, preoperative treatment option (average = 2.46 ± 1.21) than as a post-surgical, rehabilitation option (average = 2.30 ± 1.08, with "2" = "uncomfortable" and "3" = "somewhat comfortable" for both options). DO neurosurgeons were significantly more comfortable recommending both preoperative and postoperative care than MDs (preoperative, averages, DO = 3.71 ± 1.50 and MD = 2.38 ± 1.15, p=0.0042; postoperative, averages, DO = 3.29 ± 1.25 and MD = 2.24 ± 1.04, p=0.0122). Comparatively, both MDs and DOs were more comfortable offering OMM as a pain management option (average = 2.68 ± 1.14, with "2" = "uncomfortable" and "3" = "somewhat comfortable"), with DOs again showing a significantly higher degree of comfort than MDs (averages, MD = 2.60 ± 1.11 vs. DO = 4.00 ± 0.82, p=0.0014) (Figure 1C).

The survey data showed a clear interest in seeing more research into potential applications of OMM in the field of neurosurgery, with nearly equal interest between MDs and DOs. If presented with research demonstrating positive applications of OMM relevant to the management of neurosurgery patients, there was a high interest in integrating those applications into their practice. Although not statistically significant, there was a slightly higher level of interest among MDs than DOs (averages, MD = 3.83 ± 0.89, DO = 3.43 ± 1.51, p=0.2744, where "3" = "somewhat interested" and "4" = "interested") (Figure 1D).

**Figure 2:**
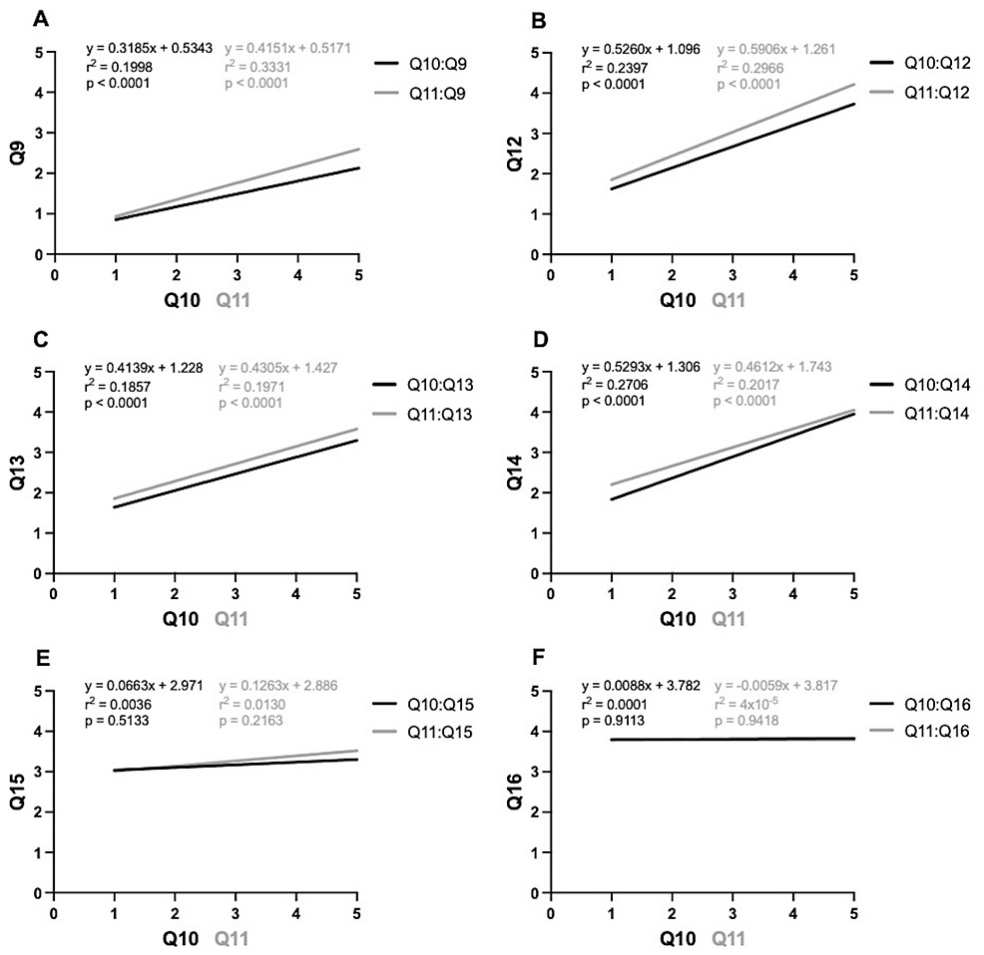
Correlations between survey data (A) Greater degrees of familiarity with the tenets of osteopathic medicine, as well as familiarity with the applications of OMM, were positively correlated with the number of times neurosurgeons either use OMM on their patients or refer their patients specifically for OMM. (B-D) There is a positive correlation between familiarity with the tenets and applications of OMM and neurosurgeons’ level of comfort in recommending OMM for their patients. (E-F) There is no correlation between the level of familiarity with osteopathic tenets and neurosurgeons’ interest in seeing more osteopathic research targeted to the field of neurosurgery or their interest in applying OMM to their practice. Statistical significance was defined as p<0.05.

The reported levels of familiarity with the tenets of osteopathic medicine and potential applications of OMM in practice were positively correlated with the number of referrals neurosurgeons gave their patients for OMM (p<0.0001) (Figure 2A). Greater reported familiarity with the tenets of osteopathic medicine and applications of OMM showed a positive correlation with neurosurgeons' comfort in recommending OMM as a nonsurgical, preoperative treatment option (p<0.0001) (Figure 2B), as a post-surgical, rehabilitative treatment option (p<0.0001) (Figure 2C), and as a pain management option (p<0.0001) (Figure 2D). However, no significant correlation was observed between these levels of familiarity and the desire to see more osteopathic-based neurosurgery research (Figure 2E) or the inclination to incorporate OMM into their practice if presented with research-based evidence showing clinical benefit (Figure 2F). In fact, both MDs and DOs expressed interest in seeing further osteopathic-based neurosurgery research (averages, MD = 3.14 ± 1.21, DO = 3.14 ± 1.68, p<0.9999) and trended toward likely integration of OMM into their practice if presented with research proving clinical benefit to their patients (averages, MD = 3.83 ± 0.89, DO = 3.43 ± 1.51, p<0.2744), irrespective of their understanding of the principles and applications of OMM (Table 3) [36,37].

**Table 3:** Survey question data (questions 9-16) The data reported in the columns labeled "Total," "MD," and "DO" is the mean ± the standard deviation (SD). The final column is the statistical significance of the difference between the data reported in the "MD" and "DO" columns as determined via a two-tailed T-test. Statistical significance was defined as p<0.05.

Survey question	Total	MD	DO	MD vs. DO
Q9: About how often do you use, or refer a patient for, osteopathic manipulative medicine in your practice? (1=never, 2=once per year, 3=once per month, 4=once per week, 5=every day)	1.36 (±0.76)	1.33 (±0.76)	1.86 (±1.22)	ns p=0.0910
Q10: On a scale of 1-5, what is your familiarity with the tenets of osteopathic medicine? (1=no knowledge; 2=unfamiliar; 3=somewhat familiar; 4=familiar; 5=very familiar)	2.60 (±1.12)	2.47 (±1.03)	4.57 (±0.53)	**** p<0.0001
Q11: On a scale of 1-5, what is your familiarity with applications of osteopathic manipulative techniques in your practice? (1=no knowledge; 2=unfamiliar; 3=somewhat familiar; 4=familiar; 5=very familiar)	2.03 (±1.12)	1.96 (±1.05)	3.29 (±1.38)	** p=0.0018
Q12: On a scale of 1-5, how comfortable are you recommending osteopathic manipulative medicine as a non-surgical, preoperative option (in general) for your patients? (1=very uncomfortable; 2=uncomfortable; 3=somewhat comfortable; 4=comfortable; 5=very comfortable)	2.46 (±1.21)	2.38 (±1.15)	3.71 (±1.50)	** p=0.0042
Q13: On a scale of 1-5, how comfortable are you recommending osteopathic manipulative medicine as a post-surgical, rehabilitation option (in general) for your patients? (1=very uncomfortable; 2=uncomfortable; 3=somewhat comfortable; 4=comfortable; 5=very comfortable)	2.30 (±1.08)	2.24 (±1.04)	3.29 (±1.25)	* p=0.0122
Q14: On a scale of 1-5, how comfortable are you recommending osteopathic manipulative medicine as a pain management option (in general, both pre- and post-operatively) for your patients? (1=very uncomfortable; 2=uncomfortable; 3=somewhat comfortable; 4=comfortable; 5=very comfortable)	2.68 (±1.14)	2.60 (±1.11)	4.00 (±0.82)	** p=0.0014
Q15: On a scale of 1-5, how interested are you in seeing more research into potential applications of osteopathic manipulative medicine in the field of neurosurgery? (1=no interest; 2=uninterested; 3=somewhat interested; 4=interested; 5=this must happen)	3.14 (±1.23)	3.14 (±1.21)	3.14 (±1.68)	ns p>0.9999
Q16: On a scale of 1-5, if you were presented with peer-reviewed research showing positive results from osteopathic manipulative techniques used in neurosurgical patient populations, how likely are you to integrate their use into your own practice? (1=I would never; 2=very unlikely; 3=unlikely; 4=likely; 5=very likely)	3.81 (±0.94)	3.83 (±0.89)	3.43 (±1.51)	ns p=0.2744

## Discussion

The findings of this survey indicate that neurosurgeons are eager to see more research into the potential applications of osteopathic medicine within their discipline, regardless of their specific medical degree. While DOs are estimated to comprise 2.0% of active physicians specializing in neurosurgery [44], 5.9% of our survey respondents were DOs, a response rate representative of nearly three times the proportion of DOs currently in the field. A significant barrier to the production of more osteopathic research, as identified by one respondent to question #17 of our survey, was a lack of local care providers qualified to perform OMM, which was a major barrier to the potential integration of OMM into their practice.

Of the 78,904 active US physicians carrying a DO degree, 116 are neurosurgeons [44]. The scarcity of osteopathically trained neurosurgeons is further reflected in the number of publications in major neurosurgery journals. Of the more than 105,000 physicians (allopathic and osteopathic) who have ever published at least one manuscript in a JNPG journal (Journal of Neurosurgery, Journal of Neurosurgery: Spine, Journal of Neurosurgery: Pediatrics, Neurosurgical Focus) since its inception in 1944, only 335 authors were osteopathic physicians [45]. Mentorship is crucial in the development of research skills and professional networks, especially in a field as highly specialized and complex as neurosurgery. The combination of a few successfully publishing osteopathic physician mentors and osteopathic physician fellows in the AANS, one of the world’s largest associations of neurosurgeons, may fail to incentivize, and may even dissuade, osteopathic physicians from contributing toward neurosurgery-focused osteopathic research in the future.

Although the current percentage of neurosurgeons carrying a DO degree is low, there are indications this may change in the near future. A report released in 2019 by the American Association of Colleges of Osteopathic Medicine (AACOM) revealed that while MDs and DOs made up 60.3% and 16.6%, respectively, of Accreditation Council for Graduate Medical Education accredited programs (international medical graduates = remaining 23.0%) [46], For the first time in history, osteopathic medical students comprise more than 25% of all US medical students. More than 7,000 osteopathic medical students graduated in 2021, and 8,945 first-year osteopathic medical students are expected to matriculate in the 2021-22 academic year, joining more than 121,000 other DOs within the United States [47]. Nevertheless, while the total number of osteopathic medical students is currently at historic highs, it has yet to be seen if this will translate to greater numbers of DO neurosurgeons in the future.

In general, DO neurosurgeons in our survey reported greater familiarity with osteopathic medicine than their allopathic colleagues. Our survey demonstrated that reported familiarity with the tenets and applications of OMM was positively correlated with neurosurgeons’ reported rate of patient referrals for OMM-specific treatment, as well as their comfort in recommending its use in perioperative settings and as a pain management strategy. One DO respondent to question #17 reported that they were "somewhat comfortable" recommending OMM as a treatment option, but cited their lack of use since medical school as their barrier to integrating it into their practice. This finding implies that enhancing neurosurgeons’ understanding of osteopathic medicine may lead to greater comfort in utilizing OMM in practice, may lead to a rise in the volume of referrals for OMM, and may lead to a general increase in rates of use of OMM in the care of neurosurgical patients.

Osteopathic physicians are trained nearly identically to their allopathic colleagues, with additional learning components of OMM accounting for the majority of the educational distinction. The curriculum of osteopathic medical students involves an additional ~200 hours of instruction in osteopathic medicine, distributed between didactic lectures and practical OMM laboratories [48]. DO students take COMLEX-USA for DO licensure, a three-level national standardized licensure examination analogous to the USMLE taken by MDs, with the inclusion of testable material in osteopathic medicine [49]. Many DO students also take the USMLE; however, no pathway exists for MD students to also sit for COMLEX. It is, therefore, possible that these barriers to maintaining the educational pathway differences between MD and DO neurosurgeons, combined with the relatively low percentage of neurosurgeons carrying a DO degree, may be a significant barrier to the utilization of OMM in practice and the conduct of research into the clinical efficacy of OMM’s in the management of neurosurgical patient populations. Nevertheless, avenues exist to expose MDs to the applications of OMM. For example, MDs enrolled in an elective course offered by osteopathic physicians in the University of Wisconsin’s Department of Family Medicine resulted in allopathic physician participants reporting statistically significant gains in their attitudes and confidence regarding OMM [50].

Given neurosurgeons’ dedication to basing clinical decisions on self-evaluation and outcomes-based research, a major step in integrating OMM into neurosurgical practice is to increase the amount of osteopathic-based research targeted toward the management of neurosurgical patients. Our survey demonstrates MD and DO neurosurgeons are interested in seeing more research into applications of OMM pertaining to their patient populations, but a disparity exists between osteopathic medicine’s contributions to health and medical research compared with our allopathic colleagues [45]. In the earliest works of Andrew T. Still, the founder of osteopathic medicine, he touched upon the importance of scientific exploration, saying the basis of osteopathic medicine is meant to be based upon “exact, exhaustive, and verifiable knowledge of the structure and function of the human mechanism” [51]. While osteopathic physicians have demonstrated this culture of research in numerous subspecialties, this focus has not translated to the field of neurosurgery.

The explanations behind the current state of osteopathic research culture are multifactorial but have been well analyzed by the osteopathic community [52-54], and current barriers in neurosurgical practice have been identified by our survey respondents. At its core, the quality, quantity, and generalizability of published osteopathic research are limited. In general, much osteopathic-based research relies heavily on pilot studies of relatively small patient sizes, with a lack of supporting studies reproducing their findings. Combined with a general lack of foundational science data, the validity of conclusions generated from osteopathic research studies is often debatable. This was evidenced by three respondents to question #17 of our survey raising concerns that OMM is “unscientific” and has a “lack of scientific construct.”

A likely contributor to the relative lack of research is funding. Colleges of Osteopathic Medicine (COM) receive only 0.1% of NIH grants and are almost entirely absent from NIH study councils and study sections. Of all clinicians who submit applications to the NIH, less than 0.4% are DOs. Of these, 11% are members of allopathic medical schools. Given this disparity, the award rate for allopathic schools is 21% higher than that submitted by COMs [55]. This was recognized by US senators in July 2022, who sent a bipartisan letter urging the NIH acting director to expand funding for osteopathic research through four key recommendations: establishing a partnership with AACOM to boost NIH funding for COMs; enhancing osteopathic representation in NIH councils and study reviewers; incentivizing principal investigators from COMs; and funding projects incorporating osteopathic philosophy and OMM. Even though the 2022 omnibus spending bill acknowledged the benefit and need of osteopathic research [56], the NIH response in August 2022 asserted that DOs “straddle the complementary, integrative health, and allopathic medical communities and have historically been connected to the National Center for Complementary and Integrative Health (NCCIH),” despite less than 2% of NIH funding at COMs coming from the NCCIH [57].

Another insidious barrier to the application of OMM in the field of neurosurgery is patient safety concerns. Five respondents to question #17 cited patient safety as their major concern in integrating OMM into their practice, with specific hesitancy in treating the cervical spine with manipulative techniques. These concerns are shared by the physicians who practice OMM, as contraindications for OMM in the cervical spine are very well characterized [9,10,12]. Further research into the efficacy of various OMM techniques can better define the safe and efficacious ways to integrate OMM into the treatment plan of neurosurgical patients, potentially increasing neurosurgeons’ comfort in recommending OMM as a treatment option for their patients.

Finally, four respondents to question #17 of our survey cited time commitment as the major reason for hesitancy in integrating OMM into their practice. The majority of surgeons’ time is spent in the operating room, documenting patient encounters, attending surgery-centered meetings, and completing administrative tasks [58], leaving little time for performing OMM. However, one respondent (an MD working in an academic institution) responded to question #17, reporting that OMM is already integrated into their sub-specialty program, indicating that integration of OMM into neurosurgical treatment plans is possible.

Limitations

The data gained from this study is based on self-reported, personal responses from members of the AANS found in the "Member Directory" tab of www.aans.org. The response rate was 1.8%. The low number of responses reflects, in part, inactive email addresses. Furthermore, while all neurosurgeons are encouraged to become members of the AANS, membership is not a strict requirement. Another factor that likely contributed to the low response rate is that many neurosurgeons may have failed to respond simply due to the volume of emails they receive and time constraints. Therefore, the opinions received may not be generalizable to the entire neurosurgical community.

## Conclusions

Neurosurgeons' reported familiarity with the tenets and applications of osteopathic medicine was positively correlated with the number of referrals neurosurgeons make for OMM. Neurosurgeons’ comfort in recommending OMM for use in preoperative and postoperative management of their patients was also correlated with a higher level of familiarity. Both MD and DO neurosurgeons express a keen interest in further investigation of the uses of OMM in their patient populations and, most importantly, report a willingness to incorporate OMM into their practice if presented with research highlighting tangible clinical benefits to their patients. Initial steps toward increasing the neurosurgical community's engagement with osteopathic-based research may be facilitated by first tackling the specific obstacles recognized by the responses to this survey.
